# Analysis of Endocrine Disrupting Pesticides by Capillary GC with Mass Spectrometric Detection 

**DOI:** 10.3390/ijerph9093166

**Published:** 2012-09-04

**Authors:** Eva Matisová, Svetlana Hrouzková

**Affiliations:** Institute of Analytical Chemistry, Faculty of Chemical and Food Technology, Slovak University of Technology in Bratislava, Radlinského 9, 81237 Bratislava, Slovak Republic; Email: svetlana.hrouzkova@stuba.sk

**Keywords:** endocrine disrupting chemicals, endocrine disrupting pesticides, ultra-trace analysis, conventional capillary gas chromatography, fast gas chromatography, mass spectrometric detection

## Abstract

Endocrine disrupting chemicals, among them many pesticides, alter the normal functioning of the endocrine system of both wildlife and humans at very low concentration levels. Therefore, the importance of method development for their analysis in food and the environment is increasing. This also covers contributions in the field of ultra-trace analysis of multicomponent mixtures of organic pollutants in complex matrices. With this fact conventional capillary gas chromatography (CGC) and fast CGC with mass spectrometric detection (MS) has acquired a real importance in the analysis of endocrine disrupting pesticide (EDP) residues. This paper provides an overview of GC methods, including sample preparation steps, for analysis of EDPs in a variety of matrices at ultra-trace concentration levels. Emphasis is put on separation method, mode of MS detection and ionization and obtained limits of detection and quantification. Analysis time is one of the most important aspects that should be considered in the choice of analytical methods for routine analysis. Therefore, the benefits of developed fast GC methods are important.

## 1. Introduction

Pesticide is a general term that includes a variety of chemical and biological products used to kill or control living organisms such as rodents, insects, fungi and plants [1]. Chemical pesticides are conventionally synthetic materials that directly kill or inactivate unwanted organisms in crops, public areas, homes and gardens and parasites in medicine [2,3]. Biopesticides are pesticides derived from natural sources like animals, plants, bacteria, and certain minerals. They are environmentally safe and non-toxic to plants and animals. However, their use is limited due to poor social awareness, comparatively lower crops yields, need for frequent applications, and the fact they are generally poorly researched. On the contrary, application of chemical pesticides has proved to be economically beneficial and has increased globally especially after the advent of “Green Revolution” of the 1960s. The productivity of crops has been increased by use of suitable pesticides. Moreover, they ensure increased food production, a safe and secure food supply, and other secondary benefits. Another important advantage is the resulting reduction in costs and labor [2,3].

Even though pesticides play a significant role in agriculture, they are among the most important environmental pollutants. This is due to their widespread presence in water, soil, the atmosphere and agricultural products. Pesticides pose a major threat, not only to living organisms, but also to the environment, especially ground and surface waters. Worldwide consumption of pesticides for agriculture use is increasing constantly [2,3,4]. In the European Union (EU) alone approximately 320,000 tons of active substances are sold every year, which accounts for one quarter of the world market [5]. Humans and wildlife are continuously exposed to a number of pesticides due to human occupations, through dietary (food, drinking water) and environmental exposure (surface water, ground water, soil, air) [2,3]. Adverse effects on human health of pesticide residues are generally known to include acute neurologic toxicity, chronic neurodevelopment impairment, possibly dysfunction of the immune, reproductive systems or cancer and many other ill effects [2,4]. 

Residues in fruit and vegetables, cereals, processed baby food and foodstuffs of animal origin are controlled through a system of statutory maximum residue limits (MRLs). MRLs are deﬁned as: “the maximum concentration of pesticide residue (expressed as milligrams of residue per kilogram of commodity (mg/kg)) likely to occur in or on food commodities and animal feeds after the use of pesticides according to good agricultural practice (GAP)” [6]. There are various organizations which set MRLs, such as the European Commission (EC), Codex Alimentarius or national governments in Australia, Canada, Japan, USA, *etc*. Individual limits for different active substance/food commodity combinations are being set. As an example around 30,000 different MRLs have been set by the EC [7]. MRLs vary ordinarily within the interval 0.0008–50 mg/kg [8], typically between 0.01 and 10 mg/kg for adult population. The lower values of MRLs are set for baby food—the EC has speciﬁed a MRL of 0.010 mg/kg [9], and the lowest levels are set for particular special residues [10]. In drinking water, the permissible maximum residue level in the EU is 100 ng/L, and in the case of some persistent chlorinated pesticides the limit is set to 30 ng/L [11]. Analyses close to these above-mentioned levels correspond to what is called ultra-trace analysis. Scientiﬁcally accepted validated analytical methods for determination of compounds at low concentration levels are essential for surveillance/compliance programs established with the terminal goal of minimizing the hazards and the risks to health and achieving more sustainable use of pesticides.

There is increasing concern about certain pesticides and other synthetic chemicals that may act as pseudo hormones which disrupt the normal function of the endocrine system in humans and wildlife [12,13]. This specific category of pollutants, known as endocrine disrupting chemicals (EDCs) or endocrine disrupters, is comprised of the compounds that may affect the normal hormonal function or possess endocrine-related functions. During the last decades the interest and concern related to endocrine disrupters among scientists, regulators and public has increased substantially. In the last years a great deal of concern has been expressed worldwide over the increasing levels of EDCs found in the environment. This anxiety is caused by the adverse effects of these pollutants on the hormone systems of humans and wildlife, even when present at levels below ppb [14]. The present safety assessments of pesticides do not take into account of possible endocrine disrupting properties of pesticides. 

This globally increased concern about EDCs has induced a need to develop highly sensitive and specific analytical tools for their determination in food, environmental and biological samples at very low concentrations. The state-of-the-art of analytical methodologies that are currently used to screen for EDCs are limited and are often based on both biological assays and chromatographic or hyphenated techniques [15]. The enzymatic methods can serve as a tool for rapid, *in situ* screening of large numbers of samples in a short period of time [16]. Biological tests are too specific to cover a wide range of different EDCs and they achieve limits of detection in µg/L [16]. Therefore, for reliable identification and quantification of compounds at ultratrace level chromatographic methods are required. GC-MS (gas chromatography-mass spectrometry) and GC-tandem MS are used for volatile or volatilizable analytes, while LC-MS/MS (liquid chromatography with tandem MS) is aimed at determining more polar and less volatile compounds [17].

This review article focuses on pesticides that act, or may act as endocrine disruptors (endocrine disrupting pesticides (EDPs)) and their analysis by capillary GC combined with MS detection in various matrices. Some space is devoted to sample preparation methods. The main part of the paper deals with published results and methods developed for EDPs residues analysis from the point of view of separation method, mode of MS detection and ionization and obtained limits of detection and quantification. The contribution to the development of analytical methods for EDPs investigation with the utilization of fast GC with MS detection is shown. The benefits of developed fast GC methods for selected EDPs over conventional GC are discussed. Examples of real-life analyses are presented.

## 2. Definition and Characteristics of EDPs

Various types of natural and synthetic chemical compounds have been identified as EDCs. They are broadly classified into several categories, such as hormones (natural and synthetic estrogens or steroids, thyroids), pharmaceuticals and personal care products, industrial chemicals, pesticides, surfactants, and others [18]. By definition adopted by European Commission, “an endocrine disrupter is an exogenous substance or mixture that alters function(s) of the endocrine system and consequently causes adverse health effects in an intact organism, or its progeny, or (sub) populations” [19]. It is important to distinguish this from a potential endocrine disrupter, which is an exogenous substance or mixture that possesses properties that might be expected to lead to endocrine disruption in an intact organism, or its progeny, or (sub) populations [19]. EDCs were defined by the United States Environmental Protection Agency (US EPA) as “an exogenous agent that interferes with synthesis, secretion, transport, metabolism, binding action, or elimination of natural blood-borne hormones that are present in the body and are responsible for homeostasis, reproduction, and developmental process“. According to Diamanti-Kandarakis *et al*. [20] it is necessary to broaden the term—an EDC is a compound, which through environmental or inappropriate developmental exposures, alters the hormonal and homeostatic systems that enable the organism to communicate and respond to environment. The European Union (EU) has done extensive work towards official designation of endocrine disrupting substances, collecting literature studies on many chemicals and establishing priority lists since the year 1999 [21]. The majority of substances registered in the list of chemicals adopted by the EC in 2007 are pesticides [22]. Pesticides are selected in Table 1, Table 2, Table 3 according to their Category based on the documented/potential endocrine effect and their chemical group.

**Table 1:** Endocrine disrupting pesticides—Category 1.

Chemical group	Pesticide	CAS	Year	Exposure
Benzoic acid derivatives	Methyl *p*-Hydroxybenzoate	99-76-3	DHI 2006	Medium
	Ethyl 4-hydroxybenzoate	120-47-8	DHI 2006	Medium
	*n*-Propyl *p*-hydroxybenzoate	94-13-3	DHI 2006	Medium
Carbamates	Carbaryl	63-25-2	BKH 2002	High
DDT derivatives and metabolites	DDT (technical), (clofenotane)	50-29-3	EM 1999	High
	*o,p’*-DDT	789-02-6	BKH 2002	High
	3-OH- *o,p’*-DDT	43216-70-2	BKH 2002	High
	4-MeO- *o,p’*-DDT	65148-72-3	BKH 2002	High
	5-OH- *o,p’*-DDT	65148-73-4	BKH 2002	High
	5-MeO- *o,p’*-DDT	65148-74-5	BKH 2002	High
	*p,p’*-DDT (clofenotane)	50-29-3	EM 1999	High
	*o,p’*-DDD	53-19-0	BKH 2002	High
	5-MeO- *o,p’*-DDD	65148-75-6	BKH 2002	High
	*p,p’*-DDD	72-54-8	BKH 2002	High
	*m,p’*-DDD	4329-12-8	BKH 2002	High
	*o,p’*-DDE	3424-82-6	BKH 2002	High
	3-MeO- *o,p’*-DDE	65148-80-3	BKH 2002	High
	4-MeO- *o,p’*-DDE	65148-81-4	BKH 2002	High
	5-MeO- *o,p’*-DDE	65148-82-5	BKH 2002	High
	*p,p’*-DDE	72-55-9	BKH 2002	High
	*o,p’-*DDA-glycinate	65148-83-6	BKH 2002	High
	*o,p’*-DDMU	14835-94-0	BKH 2002	High
	1,1,1,2-Tetrachloro-2,2-bis(4-chlorophenyl) ethane (tetrachloro DDT)	3563-45-9	EM 1999	High
	1,1,1-Trichloro-2,2-bis(4-chlorophenyl) ethane	2971-22-4	BKH 2002	High
Dicarboximides	Procymidon	32809-16-8	BKH 2002	High
	Vinclozolin	50471-44-8	EM 1999	High
Dinitroanilines	Trifluralin	1582-09-8	DHI 2006	High
Diphenyl ether	Nitrofen	1836-75-5	EM 1999	Medium
Dithiocarbamates	Mancozeb	8018-01-7	BKH 2002	High
	Maneb	12427-38-2	EM 1999	High
	Metam Natrium	137-42-8	EM 1999	High
Dithiocarbamates	Metiram (Metiram-complex)	9006-42-2	BKH 2002	High
	Thiram	137-26-8	EM 1999	High
	Zineb	12122-67-7	EM 1999	High
Formamidine	Chlordimeform	6164-98-3	DHI 2006	Low
Chlorinated Phenol	Pentachlorophenol (PCP)	87-86-5	EM 2002	High
Chloroacetanilide	Acetochlor	34256-82-1	EM 1999	High
	Alachlor	15972-60-8	EM 1999	High
Chlorophenoxy acid	2,4-dichlorophenoxybutyric acid (2,4-DB)	94-82-6	BKH 2002	-
Halogenated organic	Dibromoethane (EDB)	106-93-4	BKH 2002	Medium
	Dibromochloropropane (DBCP)	96-12-8	DHI 2006	-
HCH and isomers	Hexachlorocyclohexane	608-73-1	BKH 2002	-
	Beta-HCH	319-85-7	BKH 2002	High
	Gamma-HCH (Lindane)	58-89-9	EM 1999	High
Hydroxybenzonitrile	Ioxynil	1689-83-4	BKH 2002	Medium
Methoxychlor and derivatives	Methoxychlor	72-43-5	BKH 2002	High
	p,p’-Methoxychlor	72-43-5	BKH 2002	-
	Bis-OH-Methoxychlor	2971-36-0	BKH 2002	High
	1,3-Dichloro-2,2-bis(4-methoxy-3-methylphenyl)propane	30668-06-5	BKH 2002	-
Organochlorine	Chlordane	12789-03-6	EM 1999	High
	Chlordane ( *cis-* and *trans-*)	57-74-9	EM 1999	High
	*Cis-*Nonachlor	5103-73-1	BKH 2002	-
	Hexachlorobenzene (HCB)	118-74-1	EM 1999	High
	Kepone (Chlordecone)	143-50-0	EM 1999	High
	Mirex	2385-85-5	EM 1999	High
	Toxaphene (Camphechlor)	8001-35-2	EM 1999	High
	Trans-Nonachlor	39765-80-5	BKH 2002	-
Organotin	2-propenoic acid, 2-methyl-, methyl ester (Stannane, tributylmeacrylate)	26354-18-7	EM 1999	High
	Fentin acetate (triphenyltin acetate)	900-95-8	EM 1999	High
	Stannane, tributyl[(1-oxo-9,12-octadeca dienyl)oxy]-, (Z,Z)-	24124-25-2	EM 1999	High
	Stannane, tributyl[[[1,2,3,4,4a,4b,5,6,10,10a-decahydro-1,4a-dimethyl-7-(1-methylethyl)-1-phenanthrenyl]carbonyl]oxy]-,[1R-(1a,4ab,4ba,10aa)]-	26239-64-5	EM 1999	High
	Stannane, (benzoyloxy)tributyl-	4342-36-3	EM 1999	High
	Stannane, tributylfluoro-	1983-10-4	EM 1999	High
	Phenol, 2-[(tributylstannyl)oxy]carbony	4342-30-7	EM 1999	High
	Tributyl[(2-methyl-1-oxo-2-propenyl)oxy] stannane	2155-70-6	EM 1999	High
Organophosphorous	Fenitrothion	122-14-5	EM 1999	High
	Omethoate	1113-02-6	DHI 2006	Low
Organothiophosphor	Quinalphos (Chinalphos)	13593-03-8	DHI 2006	Medium
Pyrethroids	Bifenthrin	82657-04-3	BKH 2002	High
	Cyhalothrin	91465-08-6	BKH 2002	High
	Deltamethrin	52918-63-5	BKH 2002	High
	Resmethrin	10453-86-8	BKH 2002	High
Pyrimidines and Pyridines	Fenarimol	60168-88-9	BKH 2002	High
Pyridinecarboxylic acid	Picloram	1918-02-1	BKH 2002	Medium
Triazines and Triazoles	Amitrol (Aminotriazole)	61-82-5	EM 1999	Medium
	Atrazine	1912-24-9	EM 1999	-
	Metribuzin	21087-64-9	BKH 2002	High
	Ketoconazol	65277-42-1	BKH 2002	High
	Terbutryn	886-50-0	BKH 2002	Medium
Urea	Linuron (Lorox)	330-55-2	EM 1999	High
Other pesticides	Ethylene Thiourea (ETU)	96-45-7	DHI 2006	Low

**CAS**—chemical abstract number; **Exposure**—potential exposure to effects of pesticide on humans and wildlife, **EM 1999** (Expert Meeting)—categorization of chemicals with endocrine disrupting effects into a priority list [23]; **BKH 2002**—generation of a priority list of 553 chemicals for evaluation of endocrine disrupting properties [24]; **DHI 2006**—amendment of EDCs priority list by the study of low-production chemicals [22].

**Table 2:** Potential endocrine disrupting pesticides—Category 2.

Chemical group	Pesticide	CAS	Year
Alkyl Phthalate	Diisobutylphthalate	84-69-5	DHI 2006
Anilide	Propanil	709-98-8	EM 1999
Azole	Etridiazole	2593-15-9	BKH 2002
	Prochloraz	67747-09-5	EM 1999
	Triadimefon	43121-43-3	EM 1999
	Triadimenol	123-88-6	BKH 2002
Benzimidazole	Carbendazim	10605-21-7	EM 1999
Carbamates	Aldicarb	116-06-3	BKH 2002
	Carbofuran	1563-66-2	BKH 2002
	Fenoxycarb	72490-01-8	BKH 2002
	Methomyl	16752-77-5	BKH 2002
DDT derivatives and metabolites	*p,p’*-DDA	83-05-6	DHI 2006
Dicarboximide	Iprodione	36734-19-7	EM 1999
Dithiocarbamate	Ziram	137-30-4	EM 1999
Halogenated organic	Methyl bromide (bromomethane)	74-83-9	EM 1999
HCH isomers	Delta-HCH	319-86-8	BKH 2002
Hydroxybenzonitrils	Bromoxynil	1689-84-5	BKH 2002
Chlorinated Phenol	4-Chloro-3-methylphenol	59-50-7	EM 1999
	4-Chloro-2-methylphenol	1570-64-5	EM 1999
Chlorophenoxy acid	2,4,5-Trichlorophenoxyacetic acid (2,4,5-T)	93-76-5	BKH 2002
	2,4-Dichlorophenoxyacetic acid (2,4-D)	94-75-7	EM 1999
Organochlorine	Aldrin	309-00-2	EM 1999
	Dicofol (Kelthane)	115-32-2	EM 1999
	Dieldrin	60-57-1	EM 1999
	Endrin	72-20-8	EM 1999
	Endosulfan	115-29-7	EM 1999
	Endosulfan-alpha	959-98-8	EM 1999
	Endosulfan-beta	33213-65-9	EM 1999
	Heptachlor	76-44-8	EM 1999
	Oxychlordane	27304-13-8	EM 1999
Organophosphorous	Acephate	30560-19-1	BKH 2002
	Elsan (Dimephenthoate)	2597-03-7	DHI 2006
	Diazinon	333-41-5	EM 1999
	Dimethoate	60-51-5	EM 1999
	Chlorfenvinphos	470-90-6	BKH 2002
	Malathion	121-75-5	EM 1999
	Methylparathion	298-00-0	EM 1999
	Mevinphos (Phosdrin)	7786-34-7	BKH 2002
	Parathion (Parathion-ethyl)	56-38-2	EM 1999
	Phosophamidon	13171-21-6	BKH 2002
	Trichlorfon (Dipterex)	52-68-6	BKH 2002
Phenol	*p-*Cresol	106-44-5	DHI 2006
	4-Nitrophenol	100-02-7	DHI 2006
	*o*-Phenylphenol	90-43-7	EM 1999
Pyrethrins	Pyrethrin	121-29-9	DHI 2006
Pyrethroids	Allethrin (d-*trans*-allethrin)	584-79-2	BKH 2002
	Cypermethrin	52315-07-8	BKH 2002
	Fenothrin (sumithrin)	26002-80-2	BKH 2002
	Fenvalerate	51630-58-1	BKH 2002
	Fluvalinate	69409-94-5	BKH 2002
	Permethrin	52645-53-1	BKH 2002
Triazines	Cyanazine	21725-46-2	BKH 2002
	Prometryn	7287-19-6	BKH 2002
	Simazine	122-34-9	EM 1999
Urea	Diuron	330-54-1	EM 1999
Other pesticides	Piperonyl butoxide	51-03-6	BKH 2002
	Photomirex	39801-14-4	EM 1999

Notes: see Table 1.

**Table 3:** Pesticides with insufficient or not known endocrine disrupting effect—Category 3, sub-categories 3a, 3b.

Chemical group	Category	Pesticide	CAS No.	Year
Azoles	3b	Fenbuconazole	114369-43-6	DHI 2006
	3a	Imazalil	3554-44-0	BKH 2002
	3a	Benomyl	17804-35-2	BKH 2002
	3b	Bitertanol	55179-31-2	BKH 2002
	3b	Cyproconazole	94361-07-6	BKH 2002
Azoles	3b	Difenoconazole	119446-68-3	BKH 2002
	3b	Epiconazole	121	BKH 2002
	3b	Epoxiconazole	135319-73-2	BKH 2002
	3b	Flutriafol	76674-21-0	BKH 2002
	3b	Myclobutanil	88671-89-0	BKH 2002
	3b	Penconazole	66246-88-6	BKH 2002
	3b	Propiconazole	60207-90-1	BKH 2002
	3b	Fipronil	120068-37-3	BKH 2002
	3b	Tebuconazole	107534-96-3	BKH 2002
2,6-Dinitroanilines	3a	Oryzalin	19044-88-3	BKH 2002
	3a	Pendimethalin	40487-42-1	BKH 2002
	3b	Prodiamine	29091-21-2	BKH 2002
Formamidine	3a	Amitraz	33089-61-1	BKH 2002
Organochlorine	3a	Heptachlor-epoxide	1024-57-3	BKH 2002
Organophosphorus Phosphonoglycine Uracil	3a	Demeton-s-methyl	919-86-8	BKH 2002
	3a	Dichlorvos	62-73-7	BKH 2002
	3a	Chlorpyrifos	2921-88-2	BKH 2002
	3a	Oxydemeton-methyl	301-12-2	BKH 2002
	3a	Ronnel (fenchlorfos)	299-84-3	BKH 2002
	3a	Tetrachlorvinphos (Gardona)	22248-79-9	BKH 2002
	3b	Demefion	682-80-4	DHI 2006
	3b	Formothion	2540-82-1	DHI 2006
Amide	3a	Glyphosate	1071-83-6	BKH 2002
Aryloxyphenoxy propionic acid	3a	Bromacil	314-40-9	DHI 2006
Bipyridylium	3b	Pronamide	23950-58-5	BKH 2002
Dinitrophenol and derivatives	3b	Fluazifop-butyl	69806-50-4	BKH 2002
	3b	Paraquat	4685-14-7	BKH 2002
Dithiocarbamate Organochlorine	3b	Dinitrophenol	25550-58-7	DHI 2006
	3b	Dinoseb	88-85-7	BKH 2002
Organophosphorus	3b	Nabam	142-59-6	BKH 2002
	3b	Octachlorostyrene	29082-74-4	BKH 2002
Phosphonoglycine	3b	Glufosinate-ammonium	70393-85-0	DHI 2006
Pyridinecarboxylic acid	3b	Thiazopyr	117718-60-2	BKH 2002
Pyrethroid	3b	Esfenvalerate	66230-04-4	BKH 2002
Substituted Benzene	3b	Pentachloronitrobenzene (Quintozene)	82-68-8	BKH 2002
Tetrazine	3b	Clofentezine (chlorfentezine)	74115-24-5	BKH 2002
Thiocarbamate	3b	Molinate	2212-67-1	BKH 2002
Other pesticides	3a	Azadirachtin	11141-17-6	BKH 2002
	3a	Abamectin	71751-41-2	BKH 2002
	3a	Diphenyl	92-52-4	BKH 2002
	3a	Glufosinate	51276-47-2	BKH 2002
	3a	Chlordene	3734-48-3	BKH 2002
	3b	Dimethylformamide (DMFA)	68-12-2	BKH 2002
	3b	Ethofenprox	80844-07-1	BKH 2002

Notes: Category 3a—substances with no scientific basis for inclusion in the list according to EU; Category 3b—substances with no data available for inclusion in the list; Other notes: see Table 1.

In the United States, the US EPA has been authorized to screen all manufacturing or processing chemicals and formulations for potential endocrine activity. This was realized in a two-tiered screening and testing process, Tier 1, Tier 2. In 2009, EPA released the Final list of Chemicals for Tier 1 Screening [25], which is an update of Initial list from 2007 (some chemicals were removed). On November 2010 the US EPA published the second list of chemicals for further testing. This list of 134 chemicals includes a large number of pesticides [26]. 

Pesticides are often very persistent, with half-lifes lasting decades and are transported over long distances by global circulation. Some of the pesticides were withdrawn from general use many years ago, but are still found in the environment. For example, DDT has been found in remote areas such as the Arctic, and even in the Antarctic, even though it was banned for agricultural use in the USA in 1973 and worldwide under the Stockholm Convention [13,16]. According to Snyder and Benotti [27] who reviewed the occurrence of trace EDCs in U.S. source and finished drinking water, atrazine was present in at least 50 percent of distribution system samples at concentrations up to 50 ng/L. 

EDCs act mainly by interfering with natural hormones because of their strong potential to bind to estrogen or androgen receptors, as reviewed by Mnif *et al*. [2]. In particular, EDCs can bind to and activate various hormone receptors and then mimic the natural hormone’s action (agonist action). EDCs may also bind to these receptors without activating them. This antagonist action blocks the receptors and inhibits their action. Finally, EDCs may also interfere with the synthesis, transport, metabolism and elimination of hormones, thereby decreasing the concentration of natural hormones. For example, thyroid hormone production is known to be inhibited by ten EDPs (amitrole, cyhalothrin, fipronil, ioxynil, maneb, mancozeb, pentachloronitrobenzene, prodiamine, pyrimethanil, thiazopyr, ziram, zineb).

At the environmental level, wildlife is particularly vulnerable to the endocrine disrupting effects of pesticides. Effects linked to endocrine disruption have been largely noted in invertebrates, reptiles, fish, birds, and mammals [28]. Most of them are linked to exposure to organochlorine pesticides (OCP) and affect the reproductive function [2]. 

At the human level, EDPs have also been shown to disrupt reproductive and sexual development, and these effects seem to depend on several factors, including gender, age, diet, and occupation [2]. Age is a particularly sensitive factor. Human fetuses, infants and children show greater susceptibility than adults [29,30]. However, the effects may not become apparent until adulthood. Infants are extremely vulnerable to pre- and post-natal exposure to EDPs, resulting in a wide range of adverse health effects including possible long-term impacts on intellectual function [31] and delayed effects on the central nervous system functioning, low birth weight, fetal death and childhood cancers [32]. 

Due to the large variety of EDCs it is probable that humans and animals are exposed not to a single agent, but to a mixture of multiple endocrine disrupting agents [33]. Furthermore, the combined actions of pesticides also need to be addressed in the risk assessment process, as mixtures of these substances may cause higher toxic effects than those expected from the single compounds [2]. A central feature of endocrine disruption is that may cause detrimental effects on organisms at very low chemical concentrations [34]. Effects of EDCs at very low concentrations can be different from effects of the same chemical at higher concentrations [35]. Traditional approaches to determining safe exposure levels (for example, chemical risk assessments) do not work with EDCs.

## 3. Analysis of EDPs

The most efficient approach to pesticide analysis involves the use of chromatographic methods. Gas chromatography-mass spectrometry (GC-MS) with electron ionization (EI) and the combination of liquid chromatography (LC) with tandem mass spectrometry (LC-MS/MS) were identified as techniques most often applied in multi-residue methods for pesticides by Alder *et al.* [36]. For GC-amenable volatile and semivolatile pesticides GC methods are still preferred over LC methods due to higher resolution and lower detection limits. Especially fast GC techniques satisfy the present-day demands on faster and cost-effective analysis [4]. 

All the separate steps which make up the whole analytical method: extraction, clean up, pre-concentration, injection, separation, detection, and even data evaluation are adjusted according to the specific demands in the pesticide residues analysis. The two special essential requirements of pesticide residue analysis are the following: high sensitivity and multiresidual character of the method [4]. The tendency to reduce the absolute amount of applied pesticides and an increase of the effectiveness of EDPs at very low concentrations leads to the continuous shift to the lower analyte concentration in a sample and thereby need for methods able to reach lower limits of detection (LODs) and quantification (LOQs). Multiresidual methods provide the capability of determining different pesticide residues in a single analysis. Multiresidue procedures deal with a wide variety of physico-chemical properties of pesticides of different chemical families. The occurrence of EDCs at ultratrace concentration and with extremely diverse groups makes the analysis procedures even more challenging [18]. To overcome difficulties in the analysis, various methods have been developed, such as methods based on the use of enzymes [16], sensors and biosensors [37,38] and immunoassays [39]. However, the need for positive identification can currently be satisfied only by MS detection. Generally speaking, the mass-based methods employing mass spectrometry show relatively low detection limits as compared to other methods. For example, comparing detection limits of enzymatic methods for the detection of organochlorine, organophosphate and carbamate pesticides with chromatographic methods and MS detection it was concluded that enzymatic methods achieve limits of detection in the µg/L range, whereas traditional chromatographic methods are often able to detect pesticides at ng/L levels [16]. 

The mass-based analytical methods generally comprise a pretreatment or extraction/sample cleaning step followed by an instrumental analysis with specific settings for each target compounds based on its chemical properties. In many cases the sample preparation step plays in practice an important role in determining the overall level of analytical performance [18]. 

Several reviews have been published, which overview analytical methods for multiple classes of EDCs utilizing MS detection (GC-MS, LC-MS, GC-MS/MS, LC-MS/MS) in aqueous and solid samples by Petrovič *et al. *[40], LaFleur and Schug [41] and EDCs including pesticides in food and the environment by Holland [39], water, wastewater by Comerton *et al. *[17], and aquatic environment by Wille *et al*. [42]. At present, a combination of LC/MS and GC/MS techniques appears to be the most powerful and comprehensive approach to multi-class compound analysis as it widens the range of EDCs that can be reliable measured [17,42]. The consensus of these reviews is that mass spectrometry is undeniably the best technique to examine trace levels and provide essential identification and quantification of EDCs in food and environmental samples. Methods for analysis of EDCs residues including pesticides are reviewed also from the point of view of sample preparation [17,18,39,42]. 

The tendency towards the use of more polar and less volatile degradable pesticides has stimulated the application of LC in pesticide-residues analysis in environmental matrices with MS detection and atmospheric pressure chemical ionization (APCI) and electrospray ionization (ESI) as ionization techniques [42,43]. Matrix effects are one of the major drawbacks of LC-MS/MS, particularly when working in the ESI mode [17]. In addition, GC-MS allows less costly and easier operation than LC-MS/MS, GC/MS and GC/tandem MS generally have lower LODs when compared to LC-MS/MS [18], but requires more complicated and time-consuming sample preparation owing to the need for derivatization of polar analytes. However, commonly determined pesticides are typically volatile/semivolatile and thermostable, so GC is still a more suitable and preferable technique as reported in the literature.

### 3.1. Conventional Capillary GC with MS Detection

The present state of development of instrumentation and column technology of high-resolution GC (HRGC) offers: (i) the availability of various injection systems; (ii) accurate oven temperature control and electronic pressure control; (iii) fused silica capillary columns of different lengths, internal diameter, with the possibility to select stationary phases of different polarity and selectivity, film thickness, defined thermal stability and guaranteed reproducibility of column chromatographic properties; (iv) a number of reliable sensitive, universal and selective detectors; positive compound identification, particularly in multicomponent mixtures, is easy to establish by coupling to mass spectrometry [44]. The primary objective of chromatographic analysis is to achieve the desired resolution of compounds in the shortest possible time. Most analyses that have been performed with conventional capillary GC (columns with internal diameter, I.D., 0.20–0.32 mm) provide analysis time in the range of 10–60 min, depending on the type of sample, the number of components to be analyzed and the chosen experimental conditions.

#### 3.1.1. Sample Matrix and Sample Preparation

The choice of method for EDPs analysis will depend not only on the properties of analytes, but also on the analyzed sample matrix. In the analysis of EDP residues alone, or within multi-class EDC analysis the main interest is paid to the analysis of waters, sediments and agricultural products as fruits, vegetables and less attention is paid to the analysis of indoor dust, *etc*. (Table 4). Composition of co-extracted components depends on sample matrix and markedly influences matrix effects at the injector, column and detector site, identification and quantification of analytes. Therefore, the whole analysis must be built-up according to the matrix composition, for example the sample preparation step in food samples depends on fat content.

Sample preparation prior to injection onto the analytical instrument is a time- and labour-intensive step. Most samples are complex matrices of sludge, fats, proteins, salts, sugars, or other unwanted materials, as far as analytical determination is concerned. Ultra-trace and trace level quantitation also presents challenges, particularly when the quantity of sample available for testing is limited, e.g., biological samples.

**Table 4:** An overview of analytical methods for analysis of EDPs alone and with other groups of EDCs.

Analytes		Matrix	Sample preparation	Injection technique	LOD	Separation & detection	Ref.
23 pesticides		apples	QuEChERS	PTV, SVV	EI: 0.09–3.12 µg/kg	GC-QMS (SIM)	[45]
					NCI: 1.9–935 ng/kg	NCI, EI	
25 pesticides		apples	QuEChERS	PTV, SVV	EI: 0.02–6.32 µg/kg	fast GC-QMS (SIM)	[46]
					NCI: 0.15–619.3 ng/kg	NCI, EI	
20 OCPs		9 vegetable matrices	SBSE (PDMS 47 µL)	LVI-PTV, SVV	<10 µg/kg	GC-QMS (SIM)	[47]
						EI	
29 pesticides		fruit and vegetables	QuEChERS	PTV, SVV	≤5 µg/kg	fast GC-QMS (SIM)	[48][49]
						EI	
35 pesticides		fruit and vegetables	QuEChERS	PTV, SVV	EI: ≤5 µg/kg,	fast GC-QMS (SIM)	[50]
					NCI: ≤1 µg/kg	EI, NCI	
9 pesticides, phtalates, 1 PAH		water	on-line SPE	on-column, retaining precolumn, SVV	0.1–20 ng/L	GC-QMS (FS)	[51]
						EI	
11 pesticides, phthalates		water	on-line SPE	LVI-PTV, SVV	1–36 ng/L	GC-QMS (FS)	[52]
						EI	
HCB, atrazine, lindane, vinclozolin, malathion, aldrin, α-endosulfan, 4,4´-DDE, dieldrin, endrin, 4,4´-DDT		river water	SBSE (PDMS 63 µL)	split/splitless, LVI-PTV, SVV	0.01–0.24 µg/L	GC-QMS (FS), EI	[53]
15 herbicides, 7 OPPs, 17 OCPs		water	SBSE (PDMS 47 µL)	PTV, SVV	0.025–0.400 µg/L	GC-QMS (SIM)EI	[54]
32 EDCs and pesticides		water	SPE (LiChrolut EN/RP-18, Strata X)	splitless	5.3–95.9 ng/L	GC-MS/MS (MRM), EI, quad.,	[55]
58 potential EDCs and PPCPs (18 pesticides)		drinking water, surface, ground, waste water (raw and treated)	SPE (HLB), LLE	1. splitless2. valve	1–10 ng/L	1. GC-MS/MS, EI, IT; 2. LC-MS/MS, ESI+, ESI–, APCI, triplequad. (MRM)	[56]]
6 EDC herbicides and 3 degrade. products		natural surface water	SPE (Bond Elut-ENV)	splitless	2.3–115 ng/L	GC-QMS (SIM)EI	[57]
OPPs, OCPs, herbicides, PAHs, PCBs, phenols, organotins		estuarine and coastal water, sediments	SPE (Supelclean ENVI-18)	LVI-PTV, SVV	10–250 µg/L	GC-QMS (SIM, FS)	[15]
						EI	
1. 5 OCPs		wastewaters, surface and ground waters	SPE	1. PTV, SVV;2. valve	0.2 and 88.9 ng/L	1. GC-QMS	[58]
2. 33 multi-class pollutants						2. LC-MS/MS	
EDCs (1 pesticide), carbamazepine, pharmaceuticals		wastewater irrigated soil	ASE, isolation SPE (Oasis HLB)	splitless	0.25–2.5 ng/g	GC-QMS (SIM, FS)	[59]
						EI	
PBDEs, PCBs, insecticides, phthalates		indoor dust from vacuum cleaner	Soxhlet extraction, alumina cleaning	n. r.	3–10 ng/g	GC-QMS (SIM)	[60]
						EI	
18 OCPs		placenta samples from woman	SLE (Alumine), purification - preparative LC	n.r.	n.r.	GC-ECD	[61]
						GC-MS, IT	
dicofol, DDTs		human milk	LLE, GPC	splitless	0.1–0.2 ng/g	GC-MS (SIM)	[62]
						EI	
OCPs, PCBs		Serum samples	SPE (C18), silica gel/florisil SPE cleaning	splitless	0.4–12.0 ng/g	GC-HRMS (dual focusing sector field MS)	[63]
						(SIM), EI	
PCBs, 6 DDT metabolites, HCHs, HCB, heptachlor, chlordanes, nanochlors, mirex		blood from delivering woman	SPE (Oasis HLB), florisil SPE cleaning	splitless	n.r.	GC-MS (SIM)	[64]
						EI	
1. multiple class of pesticides		meconium	1. SPE	splitless	0.01–4.15 μg/g	GC-MS (SIM)	[65]
2. metabolites			2. derivatization , LLE			EI	

Notes: APCI—atmospheric pressure chemical ionization, ASE—accelerated solvent extraction, ECD—electron capture detector, ESI—electrospray, FS—full scan, GPC—gel permeation chromatography, HCB—hexachlorobenzene, HCHs—hexachlorocyclohexanes, HLB—hydrophilic-lipophilic balance, IT—ion trap, LLE—liquid-liquid extraction, LOD—limit of detection, LVI—large volume injection, MAE—microwave assisted extraction, MRM—multiple reaction mode, MS—mass spectrometry, MS/MS—tandem mass spectrometry, n.r.—not reported, OCPs—organochlorine pesticides, OPPs—organophosphorous pesticides, PAH—polycyclic aromatic hydrocarbon, PBDEs—pPolybrominated diphenyl ethers, PCBs—polychlorinated biphenols, PDMS—polydimethylsiloxane, PPCPs—pharmaceuticals and personal care products, PTV—programmed-temperature vaporization (injector), QMS—quadrupole MS, QuEChERS—quick, easy, cheap, effective, rugged and safe, SIM—selected ion monitoring, RRLC—rapid resolution liquid chromatography, SBSE—stir bar sorptive extraction, SLE—solid-liquid extraction, SVV—solvent vent valve.

Larger volume/quantities of sample are desirable in order to lower detection limits, but the higher the volume/quantity of sample that is pre-concentrated, the more likely the matrix will interfere [41]. Among the most discussed problems concerning analyses of pesticides are the matrix effects. To solve this problem, four types of quantitation methods can be used in pesticide residues analysis to compensate for the matrix effects [66,67,68]. For the analysis of EDCs/EDPs the same approaches to eliminate matrix effects in various matrices [17,41] are used: (i) method of standard addition [47,50,58]; (ii) use of isotopically labeled internal standards [55,56]; (iii) use of matrix-matched standards [45,46,48,49,50]; and (iv) use of analyte protectants (APs) [48,49]. For matrix-matched standardization the bracketing sequence of samples is recommended for better long term stability. Clean-up of extracts and utilization of retention gap can additionally reduce matrix effects [45,46,48,49,50,69].

Although various sample preparation techniques exist, they all have the same major objectives: isolate the component of interest from the sample; remove potential interferences from the sample matrix; if necessary, convert the analytes into a more suitable form via derivatization or pH adjustment; increase the concentration of the target analytes at concentration detectable by the selected analytical instrumentation; and provide a robust, reproducible method [17,41]. Common examples of sample preparation techniques in the literature to achieve separation of EDCs from various matrices are: liquid-liquid extraction (LLE), solid-liquid extraction (SLE) and solid-phase extraction (SPE). In the present era of “green chemistry”, the sampling preparation methods with large amounts of toxic solvents are difficult to justify for multiresidue determinations of EDCs [54]. The modern approaches are devoted to the development of a single comprehensive method utilizable for a wide variety of compounds with a single extraction in various matrices [56] or a solventless extraction technique at microscale level [47,53]. Solid-phase microextraction (SPME) and stir bar sorptive extraction (SBSE) are the often employed representatives of microextraction techniques [47,53]. The sample preparation approach known as QuEChERS, which stands for “quick, easy, cheap, effective, rugged and safe” firstly introduced by Anastassiades *et al.* [70] represents a breakthrough in the field of sample preparation. It is based on acetonitrile extraction followed by dispersive solid-phase extraction (DSPE)—all performed in two centrifugation tubes. It has been a widely used method of food sample preparation. The topic of sample preparation techniques has been addressed extensively in a number of books and reviews [71,72,73,74], therefore, they will be not discussed in detail in this review.

#### 3.1.2. GC Operating Conditions

Trace and ultra-trace concentration levels of pesticide residues in samples require non-splitting injection techniques. Splitless (SSL) [55,56,57,59,62,63,64,65], programmable temperature vaporizer (PTV) in “cold splitless” mode or “solvent vent” mode [15,45,46,47,48,49,50,52,53,54,58] are well suited (Table 4). For separation purposes, semi-polar stationary phases of analytical columns are used in general. The most used stationary phase is 5% diphenyl 95% dimethylpolysiloxane. The standard conventional column has been utilized with dimensions of 30 m × 0.25 mm I.D. × 0.25 µm for EDPs analysis (Table 4) under programmed temperature conditions. Helium is the most frequently used carrier gas. Thanks to the heteroatoms and typical functional groups a considerable portion of pesticides can be detected by selective and sensitive detectors, such as electron capture (ECD) and nitrogen phosphorous detector (NPD). For example, 15 OCPs [75] and 31 various endocrine disrupting pesticides [76] were determined in water and soil with ECD detection. Trends in GC are the ever-increasing need for positive identiﬁcation and the need for more ﬂexible systems that allows the analysis of a wide variety of samples in one system. These trends clearly result in the need of mass selective detector (MSD) [77], as it provides structural elucidation for analyte identiﬁcation. Various mass spectrometers show differences in terms of acquisition rates, detection limits, mass spectrometric resolution and quality of mass spectra obtained. Capillary GC coupled to MS detection has developed into a primary technique for identification and quantification of many EDCs using small bench-top instruments with sophisticated data systems [39]. Electron ionization is the ionization technique of the first choice. In cases requiring enhanced sensitivity and selectivity the negative/positive chemical ionization is employed [45,78]. With quadrupole mass spectrometers (QMS) for quantitation at lower trace level of pesticides selected ion monitoring (SIM) mode is typically employed, however, a part of spectral information is lost. In pesticide analysis, the detection is usually carried out through isolation of three most abundant ions [79] at the correct GC elution time. This approach is unrealistic when pesticides give less strong ions. The GC-MS/MS analysis available on triple quadrupole, ion trap and hybrid analyzers allows for more accurate analysis than GC-MS; the increased selectivity of MS/MS techniques reduces the inﬂuence of the matrix and also lowers the LODs [80]. Bench-top ion traps offer routine GC-MS/MS capability without the high cost or added complexity of multisector instrument. Similar to QMS, MS/MS detection also offers limited number of ions (consequently number of analytes), which can be recorded in one time window. Both detection methods typically have only unit mass resolution. Time of flight (TOF) instruments offer very high scan rates able to acquire complete spectra also at trace level of pesticides along with allowing the separation of overlapping peaks by automated spectral deconvolution [4]. High-resolution TOF MS is a powerful tool for reliable detection and accurate quantitation of pesticide residues even at very low concentration levels [81].

#### 3.1.3. Analytical Methods Overview—Analytes *vs.* Samples

The overview of the latest analytical methods combining sample preparation methods and GC with mass spectrometric detection for analysis of EDPs, more often EDPs within multiple classes of EDCs, in food, environmental, and biological samples is summarized in Table 4. The developed analytical methods were evaluated in terms of linearity, limits if detection and quantification, accuracy, precision, extraction efficiency, robustness of the method, screening ability, by performing several tests. Various groups of EDPs were investigated, such as carbamates, organochlorines, organophosphorous, organothiophosphates, organotins, triazines and others. Analysed samples ranged from water, sediments, soil, indoor dust, food, to biological samples. As it follows from the summarised data high resolution capillary GC coupled to mass spectrometry currently is the standard methodology for monitoring most semi-volatile EDCs, due to its versatility, high selectivity and the unequivocal spectral evidence of the individual solutes. GC-MS system can yield detection limits down to the low nanogram level, particularly if sample enrichment techniques are applied. The highest number of published papers is devoted to analysis of waters [15,51,52,53,54,55,56,57,58]. The most frequent types of samples are: river, coastal, surface, waste, ground, and drinking water. Off-line SPE was mostly applied to extract trace quantities of EDCs including pesticides employing cartridges, as octadecyl (C_18_) silica bonded phases (e.g., Supelclean ENVI-18, Lichrolut EN RP-18), polymeric sorbents (Strata X, hydrophilic-lipophilic balance polymer (HLB), styrene divinylbenzene copolymer Bond Elut-ENV) and their combinations, although the use of carbon sorbents (Envi-Carb) was reported occasionally in the literature. Their selection depends on a series of factors that include the kind of sample, sorbent selectivity required, cost, *etc*. The sample volume varied from large sample volumes (1 L) [56,57] to medium (300 mL) [58], so the enrichment factor was from 10^4^ to 10^3^. Also the large volume injection (LVI) was applied utilising PTV and QMS using SIM acquisition mode [15] to enhance sensitivity. On-line coupling of SPE with GC is complicated because it is necessary to remove traces of water before desorption into the GC column [17]. An automated on-line method was developed [51,52] to determine a group of EDCs including pesticides in several environmental water samples by preconcentrating only 15 mL of water. Pesticides LODs were 1–20 ng/L. Quantification was performed with QMS and EI in full scan (FS) mode. The other most widely used method of water sample (10/20 mL sample volume [53,54]) preparation was SBSE with polydimethylsiloxane stir bars (PDMS) and subsequent liquid desorption and LVI. The obtained LODs of pesticides were 10–240 ng/L under FS acquisition mode [53]. Multiresidue screening of EDCs in aqueous samples by multi-stir bar SBSE including *in situ* derivatization and subsequent thermodesorption was also published. In scan-mode MS, the LODs of atrazine and alachlor were 266 and 22 ng/L respectively, in SIM mode they dropped to 4.55 and 1.10 ng/L [82]. 

For solid samples such as soil and indoor dust the accelerated solvent extraction (ASE) and Soxhlet extraction with subsequent EDCs isolation by SPE and cleaning were used. It is surprising that indoor environments can be a significant source of exposure to some EDCs. Longer residence times and elevated contaminant concentrations in the indoor environment may increase the chances of exposure to these contaminants by 1,000-fold compared to outdoor exposure [60]. Pest control agents are known to be a major source of EDPs, such as pyretroids and chlordanes. Direct application, release from some household products (e.g., pest-proof wool carpet, wood), and track-in by people and pets from outside can also contribute to indoor contamination by pesticides. Exposure through ingestion and/or inhalation of indoor dust may be comparable to corresponding food consumption, especially for younger children. Method detection limits of target compounds in vacuum cleaner bags dust ranged from 3–10 ng/g. 

Biological samples of human origin were reviewed by Mniff *et al.* [2] and they comprise human tissue, human milk, serum samples, blood, *etc*. Fujii *et al*. [62] investigated the content of dicofol and DDTs in human milk. Dicofol is a potential EDPs, it is manufactured from technical grade DDT and it was confirmed to be detectable in human breast milk. Analysis of human serum samples showed the median concentrations of total OCPs to be 315 ng/g. In females, the serum concentrations of OCPs except for β-HCH were positively correlated with age, and higher values of OCPs were found in males than in females [63]. Röllin *et al*. [64] investigated the influence of environmental exposure to persistent organic pollutants in the population with the developing fetus and infants being at highest risk. DDT metabolites findings were detected in most searched participants. Ostrea *et al*. [83] searched the optimal biomarkers to detect fetal exposure to environmental pesticides by the simultaneous analysis of maternal hair and blood and infant cord blood, infant hair and meconium. Analysis of meconium was the most sensitive measure of exposure to pesticides. 

The contribution of our research group to EDP method development in non-fatty foods was first focused on the development of the conventional GC-MS method for separation, detection and quantification of EDPs belonging to different chemical classes—organochlorines, organophosphates, pyrethroids, dicarboximides, phtalamides, dinitroanilines, pyrazoles and triazinones [45]. The developed method involves the QuEChERS sample preparation method [70], modified according to our needs and resources combined with GC equipped with a PTV injector and QMS. Two ionization techniques, EI and NCI (negative chemical ionization) were utilized and compared. Better results were obtained using NCI (with methane as reagent gas) with respect to the linearity of calibration (coefficient of determination, R^2^), lowest calibration levels (LCLs), LODs and LOQs and repeatability of all measurements. LODs of all pesticides in fruit matrices varied from 0.0019 to 0.94 µg/kg for NCI and from 0.09 to 3.12 µg/kg for EI mode. 

**Figure 1:**
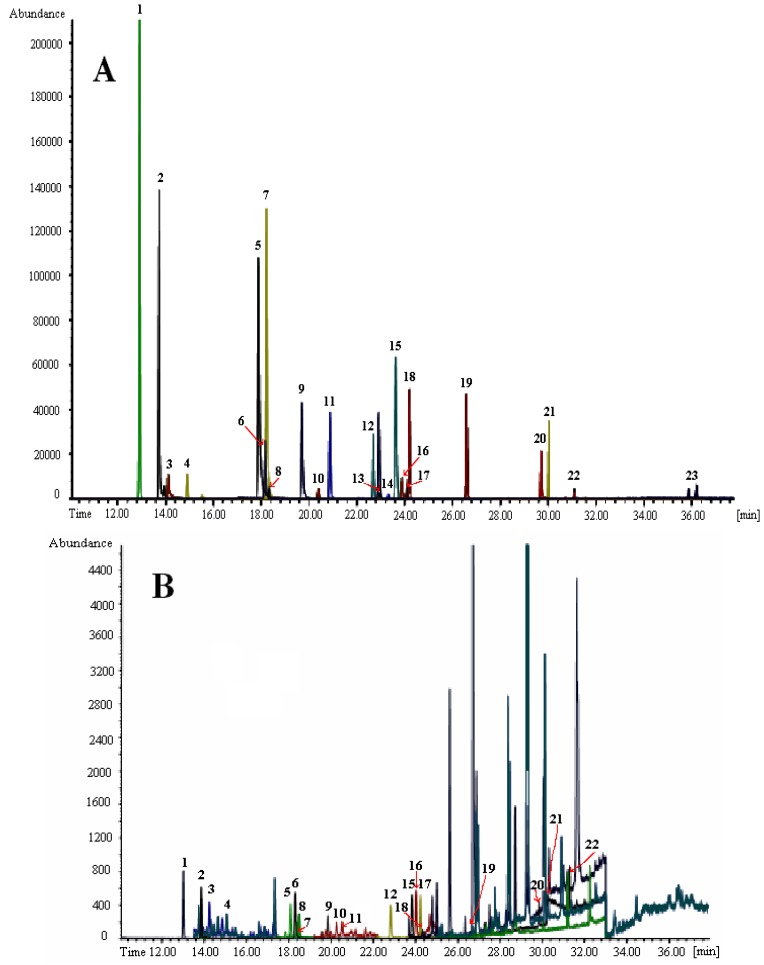
Chromatograms of target ions of 23 endocrine disrupting pesticides analyzed by capillary GC-MS in SIM mode on the conventional column HP-5 MS, 30 m × 0.25 mm I.D. × 0.25 µm connected to non-polar deactivated precolumn (1 m × 0.32 mm I.D.) in matrix-matched standard solutions at the concentration level of each analyte 10 ng/mL (corresponding to 10 µg/kg): A—NCI mode; B—EI mode [45].

To illustrate the matrix phenomena, chromatograms of the target ions of the EDPs analyzed in s real apple sample extract at the concentration level 10 ng/mL (corresponding to 10 µg/kg in fruit sample) using both MS ionization techniques in the SIM mode are presented in Figure 1. 

In the NCI mode a clean chromatogram of the target ions of EDPs without interfering peaks from matrix can be seen (Figure 1A). In the EI mode, the pesticide peak shapes are complicated due to interfering peaks of the matrix which creates problems in evaluation of the chromatograms. An important negative consequence of interfering peaks from the matrix compounds is a decreased signal to noise ratio in the EI mode. This results in a decreased response (decreased sensitivity) of the pesticides in comparison to the NCI mode at the same concentration (Figure 1B).

### 3.2. Fast Capillary GC with MS Detection

A faster GC analysis would provide unquestionable beneﬁts compared to conventional GC, such as higher laboratory throughput, reduced GC operating costs, and better analytical precision thanks to the possibility of doing more replicate analyses. There is a number of ways to increase the speed of capillary GC analysis as summarized in previous reviews [4,44,84]. Nowadays, fast GC can be performed on commercial gas chromatographs, which are equipped with standard high-speed injection systems, electronic gas pressure controls, rapid oven heating/cooling and fast detection [4]. An approach utilizing narrow-bore columns for pesticide residues analysis was developed in our research group. Fast separation with narrow-bore capillary columns as a way to reduce the run times provides separation efficiency comparable or even higher than conventional capillary columns [4,44,85]. The wide majority of high-speed GC applications described in the literature have been carried out by means of reduced I.D. columns [86]. The reduction of column I.D. is usually combined with other strategies, such as changing column geometry (column shortening approach and thinner stationary phase), or its operating parameters (higher heating rates, above optimum carrier gas flow rate and in some cases usage of hydrogen as carrier gas) that corresponds to the theoretical concept for the practical optimization of analysis speed of routine fast GC proposed by Klee and Blumberg [87]. For a number of reasons (such as sample capacity, inlet pressure values required, temperature-programmable rates), 0.1 mm I.D. columns seem to represent the current limit for columns used for routine analysis [44]. The properties of two narrow I.D.s of 0.15 and 0.1 mm (15 m length and 0.15 µm ﬁlm thickness, respectively, 10 m length and 0.10 µm ﬁlm thickness) were compared by Dömötörová *et al*. [88] with regards to their advantages, practical limitations and applicability for fast GC on commercially available instrumentation. The two columns have the same phase ratio and the same separation power. The 0.1 mm I.D. column reduces analysis time by a factor of 1.74 and signiﬁcantly narrows the peaks. The use of 0.15 mm I.D. column achieved more efficient sample transfer from inlet to the column, better sample capacity (three times higher for 0.15 mm than for 0.10 mm I.D. column) which resulted in improved ruggedness (up to 450 matrix-matched standard injections with acceptable performance of analytical column [89]) and simpler fast GC-MS method development. 

For proper operation of any “fast” column the peak broadening caused by extra-column effects must be small enough to preserve the column efficiency. This places requirements on injection and detection devices. PTV in “solvent vent” mode was shown to be very useful [4]. PTV provides the best protection against the effects of co-extracted components [66]. The two most important features of detectors combined with fast GC on narrow-bore columns are: minimal contribution to peak broadening to preserve the column efficiency and sampling frequency high enough to provide sufficient number of data points across the narrow peak for the accurate representation of the peak. The area of fast GC-MS has been reviewed and discussed in detail by a few authors [84,90,91,92]. The most important detection technique in fast GC pesticide residues analysis is EI-MS. Preferred detection system is single QMS. Possibilities and limitations of QMS in the qualitative and quantitative measurements of pesticides with fast GC separation were evaluated by Kirchner *et al.* [93] using a 0.15 mm I.D. narrow-bore capillary column. For quantitative analysis SIM is able to acquire the sufficient number of data-points for the proper peak shape reconstruction and good repeatability of peak area measurements. 

**Figure 2:**
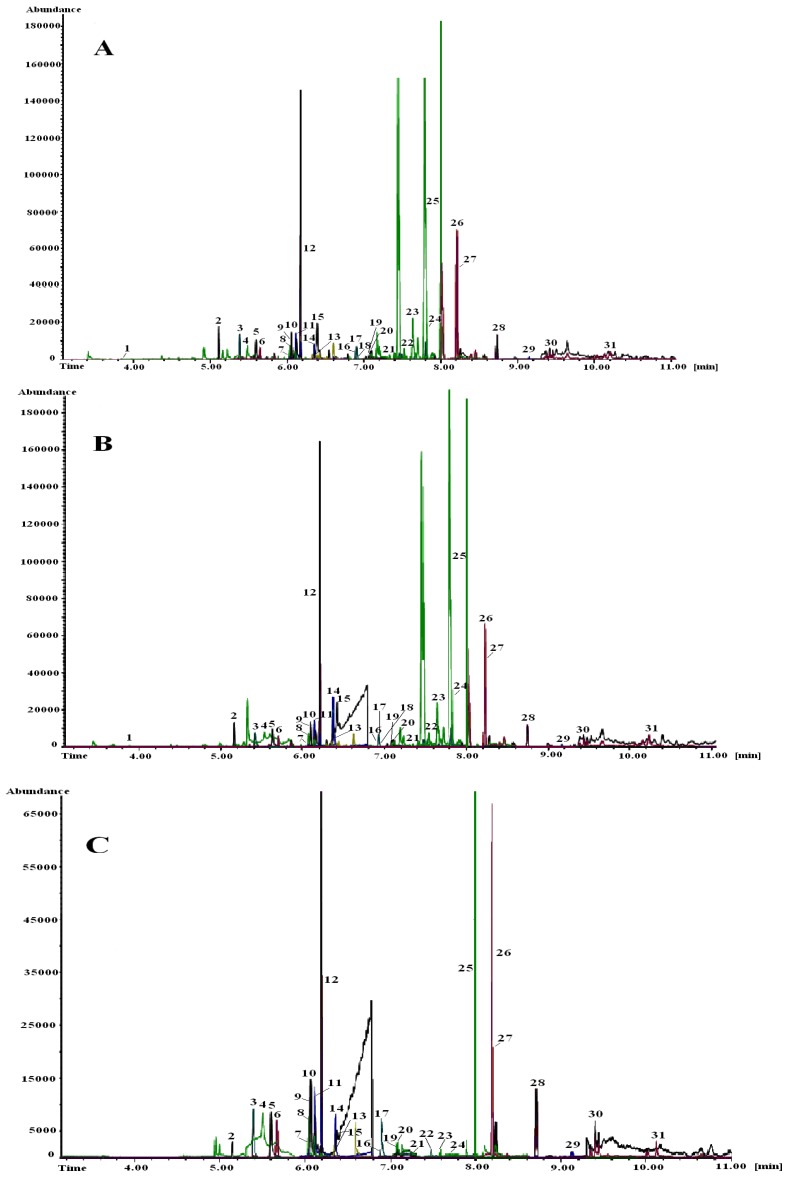
Chromatograms of target ions of 29 endocrine disrupting pesticides on the narrow-bore column CP-Sil 5 CB, 15 m × 0.15 mm I.D. × 0.15 µm connected to non-polar deactivated precolumn (1 m × 0.32 mm I.D.) in various standard solutions (50 ng /mL, corresponding to 50 µg/kg) analyzed by fast GC-MS in SIM mode: **A**—matrix-matched standard solution without APs; **B**—matrix-matched standard solution with APs; **C**—MeCN standard solution with APs [48].

The benefits of the developed fast GC-MS methods with narrow-bore column for selected EDPs in non-fatty food compared to conventional GC [45] was published by Húšková *et al.* [46,48] and Hrouzková *et al.* [50]. The QuEChERS method was used for preparation of extracts of fruit and vegetables. Bench top QMS detector in EI/NCI mode (both techniques used SIM mode) was utilised for ultratrace analysis. PTV in solvent vent mode and narrow-bore column (15 m × 0.15 mm I.D. × 0.15 µm of 5% diphenyl 95% dimethylpolysiloxane stationary phase) were used for effective and fast separation. The fast GC-MS-EI method for the determination of 29 pesticides proved or suspected to be EDCs was developed and validated [48]. LOQs were obtained in the range of 0.04 to 10 µg/kg for the majority of analytes. The method was used for evaluation of different calibration approaches: matrix-matched standard calibration and use of APs. Analyte protectants should protect co-injected analytes against degradation, adsorption, or both in the GC system. The novel concept idea was to add analyte protectants to sample extracts as well as to matrix-free (solvent) standards to induce an even response in both instances [94]. For illustration chromatograms of target ions of EDCs pesticides in various standards solutions: matrix (apple sample extract) matched standards without APs, matrix matched standards with APs and neat solvent (acetonitrile (ACN)) with APs analyzed by fast GC-MS in SIM mode at the concentration level of 50 ng/mL (corresponding to 50 µg/kg) are presented in Figure 2. Separation of 29 pesticides belonging to various chemical classes took 11 mins. The main contribution of APs approach to higher analysis speed is the time saving by easier preparation of calibration standards—furthermore only one standard is used for every matrix. Utilization of pesticide standards in a neat solvent with addition of APs provided higher values of LODs and LOQs, particularly for the most volatile and problematic analytes. Calibration with matrix-matched standards—at present the most widely used in laboratories-provided the best results in terms of measurements of linearity, instrumental LODs, LOQs and repeatability of measurements. Adverse effects caused by matrix coextractives, which result in worse precision and accuracy of analytical results were further studied by Hercegová *et al.* [49]. Sample extracts of various commodities (apple, peach, cucumber, cauliflower) were evaluated by gravimetric analysis to compare co-extracted compounds and matrix extracts background was measured in FS and SIM mode and compared. In order to evaluate the accuracy of quantitative measurements of simulated samples extracts with addition of pesticides at the concentration level of 50 µg/kg were performed utilising above mentioned different calibration standards. For the majority of the pesticides under study significant overestimation of concentration in all tested matrices was observed utilizing standards in ACN with addition of APs. The overestimation was matrix and analyte dependent and influenced by the number of injections performed. The maximum value of error of determination of average concentration was found to be 39.8%. In some cases also underestimation of quantity was observed. 

A combination of fast GC with narrow-bore column (0.15 mm I.D.) and QMS in NCI mode was introduced by Húšková *et al*. [46] and utilized for the ultratrace analysis of 25 selected EDPs. The observed pesticides were from different chemical classes (organochlorines, organophosphates, pyrethroids, dicarboximides, 2,6-dinitroanilines, triazinones, substituted ureas, phthalamides, cyclo-dienes, triazoles, imidazoles). A comparative study with EI was also carried out. Non-fatty food matrices (fruit and vegetables) were investigated. Very good results were obtained for the characterization of fast GC-NCI-MS method analysing EDPs. Instrument LODs and LOQs were found to be at pg/mL level and for the majority of analytes were up to three orders of magnitude lower for NCI compared to EI. In both ionization modes repeatability of measurements expressed as relative standard deviation (RSD) was less than 10%, which is in very good agreement with the criterion of EU. By changing from EI mode to NCI the selectivity was increased, and the measured sensitivity of the selected analytes was enhanced. Comparison of relevant validation parameters is given in Table 5.

**Table 5:** Comparison of validation parameters for NCI *vs*. EI mode of fast GC-MS analysis of EDPs residues in apple matrix [21].

Method/Results	GC-NCI-MS	GC-EI-MS
LCLs	0.01, 0.05 µg/kg	1 µg/kg
R^2^	0.9936–1.0000	0.9882–0.9999
LODs	0.15–88.82 ng/kg	0.01–6.32 µg/kg
LOQs	0.52–291.35 ng/kg	0.04–21.07 µg/kg

Notes: LCLs—lowest calibration level, R^2^—coefficient of determination, LODs—limits of detection, LOQs—limits of quantification.

## 4. Real-Life Samples Analysis

In order to assess the applicability of the developed method, several real-life samples analyses usually follow the method validation or the developed method is applied in a monitoring survey presented in a separate subsequent publication. Target compounds in the analysis were pesticide residues, or multicomponent multiclass residues of EDCs including pesticides. We present examples of occurrence of EDPs in various matrices of real samples analyzed by conventional and fast capillary GC with MS detection. In water samples, wastewaters, surface and ground waters, according to Baugros *et al*. [58] banned OCPs, DDT and its residues, and methoxychlor are still present in surface and ground waters at the detection limits, or between 0.084 and 0.302 µg/L. Domestic use pesticides, like dimethoate at concentrations 0.0021–0.0198 µg/L and propoxur 0.0045–0.0897 µg/L, respectively, were determined in wastewater treatment plant effluents. Among fungicides, tebuconazole was present in the majority of samples, even in groundwater at concentrations from 0.0258–0.8947 µg/L. Snyder and Benotti [27] published results on a U.S. drinking water quality. They surveyed the occurrence of 36 pharmaceuticals and EDCs in the source (n = 20) and finished drinking water (n = 20). Twelve compounds were detected in at least 50% of source water samples and among EDPs atrazine (28 ng/L), metalochlor (15 ng/L), TCEP (13 ng/L), triclosan (1.9 ng/L), trimethoprim (2.2 ng/L) were found (median concentrations are given in brackets). Eight compounds were detected in at least 50% of the finished drinking water samples, and atrazine, metalochlor and triclosan were confirmed among the EDPs there. 

Lopez-Espinosa *et al*. [61] analysed OCPs in 150 placenta samples from Southern Spain. Residues of one or more pesticides were detected in all of samples, with a mean of eight pesticides per placenta (range, 3–15). The highest concentrations were those of *p,p´-*DDT (2.37 ng/g of placenta), isomers and metabolites, followed by endosulfans. Endosulfan isomers and metabolites were found in more than 98% of the searched series of placentas. Lindane was detected in 74.17% of the placenta samples. With respect to the aldrin group, the order of their frequency was endrin < aldrin < dieldrin. 

**Figure 3:**
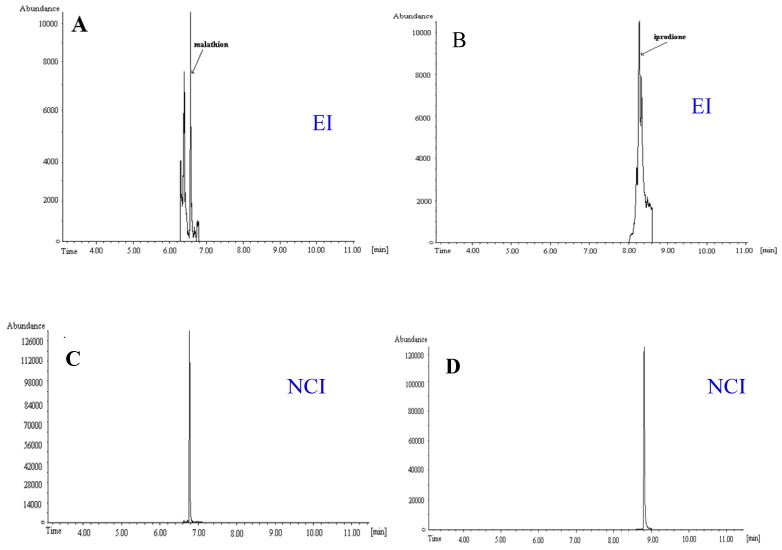
Chromatogram of target ions of EDPs analyzed by fast GC-MS in SIM in real samples; **A**—orange (malathion, determined concentration 50.1 µg/kg) in EI; **B**—strawberry (iprodione, determined concentration 40.9 µg/kg) in EI; **C**—orange in NCI; **D**—strawberry in NCI mode [50].

To show the potential of fast GC-QMS in the EI mode for the utilization in the ultratrace analysis of pesticide residues with endocrine disruption behaviour, a survey of EDPs in non-fatty food was published by Hrouzková *et al*. [50]. An important objective was to assess the occurrence of pesticides from different chemical classes suspected or known to act as endocrine disrupters in fruit and vegetable samples available on the market in Slovakia. Thirty-four samples of 20 different commodities were analyzed. Twenty-one compounds at concentrations in the range of 0.003–2.14 mg/kg were detected in 28 samples. The MRL value was exceeded in the case of dimethoate (peachA). In the case of fenitrothion (peachB) the determined concentration was at the MRL level. Seven samples contained residues of three or more EDPs. The concentrations of EDPs residues determined also in the NCI mode were in a good agreement with EI mode (Table 6). The example of pearA and kohlrabi shows the ability of the NCI-MS method for the pesticide residue analysis at the low concentration level. The given pesticides were not detected in EI mode. For illustration, the chromatograms of the target ions of EDPs residues analyzed by fast GC-MS in the SIM mode in the real sample extracts, in NCI and EI ionization modes, are given in Figure 3 [50]. It is evident, that NCI mode produces very “clean” chromatograms with high responses of analytes without influence from the matrices (orange, strawberry) as compared to results using EI mode. 

**Table 6:** Concentration *c_i_* (µg/kg) of EDPs residues in real samples and repeatability of measurements expressed as relative standard deviation RSD (%) of parallel extractions [50].

Matrix	Pesticide	NCI		EI	
		*c_i_* (µg/kg)	RSD (%)	*c_i_* (µg/kg)	RSD (%)
orange	malathion	50.1	0.52	52.5	3.5
lettuce	iprodione	40.1	1.2	42.0	3.0
pear *_A_*	iprodione	40.1	1.2	41.3	3.4
pear *_B_*	bifenthrin	69.0	5.2	64.8	4.1
	myclobutanil	0.07	6.8	n.d.	-
kohlrabi	metribuzin	0.06	3.0	n.d.	-
	vinclozolin	0.15	2.1	n.d	-
	myclobutanil	0.25	3.6	n.d.	-
plum	iprodione	234.3	0.31	241.1	2.8
strawberry	iprodione	40.9	1.2	41.3	3.4
pepper	myclobutanil	24.3	6.3	30.4	4.2
	cypermethrin	47.2	2.2	54.9	6.0

## 5. Conclusions

Many chemical pesticides are in use today and new ones are being developed. They contaminate the food chain and the environment, through groundwater, rivers and soil. Pesticides are intentionally toxic towards target pests, but unintentionally toxic towards humans and wildlife. Unintentional health effects of pesticides include their endocrine disrupting effects. EDPs are known as a class of EDCs of xenobiotic origin. The significance and importance of research on analysis methods for EDCs/EDPs and the current state of EDPs are discussed in the paper. The concentration of a single EDC, or mixture of EDCs may be surprisingly low, in some instances less than 1 ng/L in aqueous system [41]. These findings are driving the detection limits of the analytical methods used to monitor EDCs/EDPs lower than they have ever been before. A variety of sample preparations and analytical techniques that offer ultra-trace detection limits were investigated (Table 4). The use of chromatographic methods hyphenated with mass-spectrometric detection provide the excellent sensitivity and precision. GC-MS remains the most useful and sensitive method for the detection of trace levels of volatile EDCs/EDPs. The most often used MS detector has been the quadrupole MS with EI. By utilising tandem MS it is possible to reach even lower detection limits. The powerful technique GC-TOF-MS provides the the possibility of performing untargeted fast GC of pesticides in complex samples on commercial instruments. At present a combination of GC-MS and LC-MS/MS techniques appears to be the best approach to multi-compound class analysis [17]. 

The main part of the review paper is devoted to the contribution of capillary GC-MS for method development for EDP analysis with the utilization of conventional and fast GC. The search for different calibration approaches based on the matrix-matched standardization, the application of analyte protectants and the influence of different matrices with differing amounts of co-extractants was studied with the aim of eliminating the adverse effects caused by matrix interferences. According to several authors [47,58] the use of the standard addition method is the best approach to solving matrix effect phenomena. Isotopically labelled surrogate and internal standard compounds can also be used to compensate for matrix effects [17,55,56]. The combination of conventional and efficient fast GC separation and selective MS detection with NCI results to selectivity enhancement and decrease of the limits of detection and quantification.

For EDP residues analysis ultrasensitive analytical methods are required and there is still the need to improve the performance and ruggedness of analyses. Despite the significant progress in the analytical instrumentation development, for most of analytes/matrices combinations there is a continuous need to employ effective extraction and preconcentration methods to reach the ultra-trace concentration levels and to avoid adverse effects of the matrix co-extractants on the results of quantitative analysis and maintenance of GC instrumentation. Identification and determination of endocrine disrupting pesticides is a relevant research trend and a source of progress of analytical methods as a base for necessary changes in regulations of the quality of food and environment in the future is expected.

## References

[1] National Pesticides Strategy: Part 1: The Background and the Need for Change. http://www.pesticides.gov.uk/environment.asp?id=1523.

[2] Mnif W., Hassine A.I.H., Bouaziz A., Bartegi A., Thomas O., Roig B. (2011). Effect of endocrine disruptor pesticides: A review. Int. J. Environ. Res. Public Health.

[3] Bhadekar R., Swanandi P., Tale V., Nirichan B. (2011). Developments in analytical methods for detection of pesticides in environmental samples. Am. J. Anal. Chem..

[4] Dömötörová M., Matisová E. (1207). Fast gas chromatography for pesticide residues analysis. J. Chromatogr. A.

[5] Proposed PAHO/WHO Plan of Action for Technical cooperation in Food Safety, 2006-2007. ftp://ftp.fao.org/docrep/fao/meeting/010/af272e.pdf.

[6] Maximum Residue Levels. http://www.pesticides.gov.uk/guidance/industries/pesticides/advisory-groups/PRiF/PRC+%28Pesticides+Residues+Commitee%29/About+pesticides+in+food/maximum-residue-levels.

[7] UK Pesticides Strategy: A Strategy for the Sustainable Use of Plant Protection Products, Department of Environment, Food and Rural Affairs, London, United Kingdom. http://www.pesticides.gov.uk/Resources/CRD/Migrated-Resources/Documents/U/Updated_National_Strategy.pdf.

[8] Status of Active Substances under EU Review (doc. 3010). http://ec.europa.eu/food/plant/protection/evaluation/stat_active_subs_3010_en.xls.

[9] (2003). Commission Directive 2003/13/EC of 10 February 2003 amending Directive 96/5/EC on Processed Cereal-Based Foods and Baby Foods for Infants and Young Children. http://eur-lex.europa.eu/LexUriServ/LexUriServ.do?uri=OJ:L:2003:041:0033:0036:EN:PDF.

[10] (2003). Commission Directive 2003/14/EC of 10 February 2003 amending Directive 91/321/EEC on Infant Formulae and Follow-On Formulae. http://eur-lex.europa.eu/LexUriServ/LexUriServ.do?uri=OJ:L:2003:041:0037:0040:EN:PDF.

[11] Council Directive 98/83/EC of 3 November 1998 on the Quality of Water Intended for Human Consumption. http://eur-lex.europa.eu/LexUriServ/LexUriServ.do?uri=OJ:L:1998:330:0032:0054:EN:PDF.

[12] Colborn T., vom Saal F.S., Soto A.M. (1993). Developmental effects of endocrine-disrupting chemicals in wildlife and humans. Environ. Health Perspect..

[13] Lintelmann J., Katayama A., Kurihara N., Shore L., Wenzel A. (2003). Endocrine disruptors in the environment. Pure Appl. Chem..

[14] Jobling S. (2004). Endocrine disruption in wild fish. Pure Appl. Chem..

[15] Almeida C., Serôdio P., Florencio M.H., Nogueira J.M.F. (2007). New strategies to screen for endocrine-disrupting chemicals in the Portuguese marine environment utilizing large volume injection-capillary gas chromatography-mass spectrometry combined with retention time locking libraries (LVI-GC-MS-RTL). Anal. Bioanal. Chem..

[16] Van Dyk J.S., Pletschke B. (2011). Review on the use of enzymes for the detection of organochlorine, organophosphate and carbamate pesticides in the environment. Chemosphere.

[17] Comerton A.M., Andrews R.C., Bagley D.M. (2009). Practical overview of analytical methods for endocrine-disrupting compounds, pharmaceuticals and personal care products in water and wastewater. Philos. Trans. R. Soc. A.

[18] Chang H.S., Choo K.H., Lee B., Choi S.J. (2009). The methods of identification, analysis, and removal of endocrine disrupting compounds (EDCs) in water. J. Hazard. Mater..

[19] Endocrine Disruptors Website. http://ec.europa.eu/environment/endocrine/definitions/endodis_en.htm.

[20] Diamanti-Kandarakis E., Bourguignon J.P., Giudice L.C., Hauser R., Prins G.S., Soto A.M., Zoeller R.T., Gore A.C. (2009). Endocrine-disrupting chemicals: An endocrine society scientific statement. Endocr.Rev..

[21] Hrouzková S., Matisová E., Soundararajan R.P. (2012). Endocrine Disrupting Pesticides. Pesticide-Advances in Chemical and Botanical Pesticides.

[22] Commission of the European Communities: Commission Staff Working Document on the Implementation of the “Community Strategy for Endocrine Disrupters” SEC 2007. http://ec.europa.eu/environment/endocrine/documents/sec_2007_1635_en.pdf.

[23] European Commission: Community Strategy for Endocrine Disrupters. http://ec.europa.eu/environment/docum/99706sm.htm.

[24] European Commission Commission Staff Working Document on the Implementation of the “Community Strategy for Endocrine Disrupters”**-**A Range of Substances Suspected of Interfering with the Hormone Systems of Humans and Wildlife (COM (1999) 706), (COM (2001) 262) and (SEC (2004) 1372). http://ec.europa.eu/environment/endocrine/documents/sec_2007_1635_en.pdf.

[25] Final List of Initial Pesticide Active Ingredients and Pesticide Inert Ingredients to be Screened under the Federal Food, Drug, and Cosmetic Act. http://www.epa.gov/scipoly/oscpendo/pubs/final_list_frn_041509.pdf.

[26] US Environmental Protection Agency (2010). Endocrine disruptor screening program; Second list of chemicals for tier 1 screening. Fed.Regist..

[27] Snyder S.A., Benotti M.J. (2010). Endocrine disruptors and pharmaceuticals: Implications for water sustainability. Water Sci. Technol..

[28] Mnif W., Pillon A., Balaguer P., Bartegi A. (2007). Endocrine xenoestrogenics disrupters, molecular methods and detection methods. Therapie.

[29] Birnbaum L.S., Fenton S.E. (2003). Cancer and developmental exposure to endocrine disruptors. Environ. Health Perspect..

[30] Sharpe R.R.M. (2006). Pathways of endocrine disruption during male sexual differentiation and masculinisation. Best Pract. Res. Clin. Endocrinol. Metab..

[31] Eskenazi B., Marks A.R., Bradman A., Fenster L., Johnson C., Barr D.B. (2006). *In utero* exposure to dichlorodiphenyltrichloroethane (DDT) and dichlorodiphenyldichloroethylene (DDE) and neurodevelopment among young Mexican American children. Pediatrics.

[32] Ribas-Fito N., Cardo E., Sala M., Eulalia de Muga M., Mazon C., Verdu A., Kogevinas M., Grimalt J.O., Sunyer J. (2003). Breastfeeding exposure to organochlorine compounds and neurodevelopment in infants. Pediatrics.

[33] Lagana A., Bacaloni A., de Leva I., Faberi A., Fago G., Marino A. (2004). Analytical methodologies for determining the occurrence of endocrine disrupting chemicals in sewage treatment plants and natural waters. Anal.Chim. Acta.

[34] Fang H., Tong W., Shi L.M., Blair R., Perkins R., Branham W., Hass B.S., Xie Q., Dial S.L., Moland C.L., Sheehan D.M. (2001). Structure-activity relationships for a large diverse set of natural, synthetic, and environmental estrogens. Chem. Res. Toxicol..

[35] Colborn T. TEDX-The Endocrine Disruption Exchange,Endocrine Disruption Fact Sheet. http://www.endocrinedisruption.com/files/EDFactSheet11-7-11.pdf.

[36] Alder L., Greulich K., Günther K., Kempe G., Bärbel V., Vieth B. (2006). Residue analysis of 500 high priority pesticides: Better by GC-MS or LC-MS/MS?. Mass Spectrom. Rev..

[37] Bezbaruah A.N., Kalita H., Virkutyte J., Jegatheesan V., Varma R.S. (2010). Sensors and Biosensors for Endocrine Disrupting Chemicals: State-of-the-Art and Future Trends. Treatment of Micropollutants in Water and Wastewater.

[38] Dostálek J., Přibyl J., Homola J., Skládal P. (2007). Multichannel SPR biosensor for detection of endocrine disrupting compounds. Anal.Bioanal. Chem..

[39] Holland P.T. (2003). Analysis of endocrine active substances in food and the environment. Pure Appl. Chem..

[40] Petrovič M., Eljarrat E., López de Alda M.J., Barceló D. (2002). Recent advances in the mass spectrometric analysis related to endocrine disrupting compounds in aquatic environmental samples. J. Chromatogr. A.

[41] LaFleur A.D., Schug K.A. (2011). A review of separation methods for the determination of estrogens and plastics-derived estrogen mimics from aqueous systems. Anal.Chim. Acta.

[42] Wille K., de Brabander H.F., Vanhaecke L., de Wulf E., van Caeter P., Janssen C.R. (2012). Coupled chromatographic and mass-spectrometric techniques for the analysis of emerging pollutants in the aquatic environment. Trends Anal. Chem..

[43] Kuster M., López de Alda M., Barceló D. (1216). Liquid chromatography-tandem mass spectrometric analysis and regulatory issues of polar pesticides in natural and treated waters. J. Chromatogr. A.

[44] Matisová E., Dömötörová M. (1000). Fast gas chromatography and its use in trace analysis. J. Chromatogr. A.

[45] Húšková R., Matisová E., Švorc Ľ., Mocák J., Kirchner M. (2009). Comparison of negative chemical ionization and electron impact ionization in gas chromatography-mass spectrometry of endocrine disrupting pesticides. J. Chromatogr. A.

[46] Húšková R., Matisová E., Hrouzková S., Švorc L. (1216). Analysis of pesticide residues by fast GC in combination with negative chemical ionization mass spectrometry. J. Chromatogr. A.

[47] Barriada-Pereira M., Serodio P., Gonzalez-Castro M.J., Nogueira J.M.F. (1217). Determination of organochlorine pesticides in vegetable matrices by stir bar sorptive extraction with liquid desorption and large volume injection-gas chromatography-mass spectrometry towards compliance with European Union directives. J. Chromatogr. A.

[48] Húšková R., Matisová E., Ondreková S. (2010). Fast GC-MS of endocrine disrupting chemicals. Int. J. Environ. Anal. Chem..

[49] Hercegová A., Húšková R., Matisová E. (2010). Evaluation of different calibration approaches in pesticide residues analysis in non-fatty food using fast GC-MS. Int. J. Environ. Anal. Chem..

[50] Hrouzková S., Matisová E., Andraščíková M., Horváth M., Húšková R., Ďurčanská J.  (2012). Survey of low-level endocrine disrupting pesticides in food matrices in Slovakia. Int. J. Environ. Anal. Chem..

[51] Brossa L., Marcé R.M., Borrull E., Pocurull E. (2002). Application of on-line solid-phase extraction-gas chromatography-mass spectrometry to the determination of endocrine disruptors in water samples. J. Chromatogr. A.

[52] Brossa L., Marcé R.M., Borrull F., Pocurull E. (2003). Determination of endocrine-disrupting compounds in water samples by on-line solid-phase extraction-programmed-temperature vaporisation-gas chromatography-mass spectrometry. J. Chromatogr. A.

[53] Peñalver A., Garcia V., Pocurull E., Borrull F., Marcé R.M. (1007). Stir bar sorptive extraction and large volume injection gas chromatography to determine a group of endocrine disrupters in water samples. J. Chromatogr. A.

[54] Serôdio P., Nogueira J.M.F. (2004). Multi-residue screening of endocrine disrupters chemicals in water samples by stir bar sorptive extraction-liquid desorption-capillary gas chromatography-mass spectrometry detection. Anal.Chim. Acta.

[55] Mansilha C., Melo A., Rebelo H., Ferreira I.M., Pinho O., Domingues V., Pinho C., Gameiro P. (1217). Quantification of endocrine disruptors and pesticides in water by gas chromatography-tandem mass spectrometry: Method validation using weighted linear regression schemes. J. Chromatogr. A.

[56] Trenholm R.A., Vanderford B.J., Holady J.C., Rexing D.J., Snyder S.A. (2006). Broad range analysis of endocrine disruptors and pharmaceuticals using gas chromatography and liquid chromatography tandem mass spectrometry. Chemosphere.

[57] Nevado J.J.B., Cabanillas C.G., Llerena M.J.V., Rodriguez Robledo V. (2007). Sensitive SPE GC-MS-SIM screening of endocrine-disrupting herbicides and related degradation products in natural surface waters and robustness study. Microchem.J..

[58] Baugros J.B., Giroud B., Dessalces G., Grenier-Loustalot M.F., Cren-Olivé C. (2008). Multiresidue analytical methods for the ultra-trace quantification of 33 priority substances present in the list of REACH in real water samples. Anal.Chim. Acta.

[59] Durán-Alvarez J.C., Becerill-Bravo E., Castro V.S., Jiménez B., Gibson R. (2009). The analysis of a group of acidic pharmaceuticals, carbamazepine, and potential endocrine disrupting compounds in wastewater irrigated soils by gas chromatography-mass spectrometry. Talanta.

[60] Hwang H.M., Park E.K., Young T.M., Hammock B.D. (2008). Occurrence of endocrine-disrupting chemicals in indoor dust. Sci. Total Environ..

[61] Lopez-Espinosa M.J., Granada A., Carreno J., Salvatierra M., Olea-Serrano F., Olea N. (2007). Organochlorine pesticides in placentas from Southern Spain and some related factors. Placenta.

[62] Fujii Y., Haraguchi K., Harada K.H., Hitomi T., Inoue K., Itoh Y., Watanabe T., Takenaka K., Uehara S., Yang H.R. (2011). Detection of dicofol and related pesticides in human breast milk from China, Korea and Japan. Chemosphere.

[63] Kang J.H., Park H., Chang Y.S., Choi J.W. (2008). Distribution of organochlorine pesticides (OCPs) and polychlorinated biphenyls (PCBs) in human serum from urban areas in Korea. Chemosphere.

[64] Röllin H.B., Sandanger T.M., Hansen L., Channa K., Odland J.Ø. (2009). Concentration of selected persistent organic pollutants in blood from delivering women in South Africa. Sci. Total Environ..

[65] Bielawski D., Ostrea E., Posecion N., Corrion M., Seagraves J. (2005). Detection of several classes of pesticides and metabolites in meconium by gas chromatography-mass spectrometry. Chromatographia.

[66] Hajšlová J., Zrostlíková J. (1000). Matrix effects in(ultra) trace analysis of pesticide residues in food and biotic matrices. J. Chromatogr. A.

[67] Poole C.F. (1158). Matrix-induced response enhancement in pesticide residue analysis by gas chromatography. J. Chromatogr. A.

[68] Húšková R., Kirchner M., Matisová E. (2007). Matrix effects and its elimination in analysis of pesticide residues in food by gas chromatography. Chem. Listy.

[69] Hercegová A., Dömötörová M., Kružlicová D., Matisová E. (2006). Comparison of sample preparation methods combined with fast gas chromatography-mass spectrometry for ultratrace analysis of pesticide residues in baby food. J. Sep. Sci..

[70] Anastassiades M., Lehotay S.J., Štajnbaher D., Schenck F.J. (2003). Fast and easy multiresidue method employing acetonitrile extraction/partitioning and “dispersive solid-phase extraction” for the determination of pesticide residues in produce. J. AOAC Int..

[71] Farre M., Brix R., Kuster M., Rubio F., Goda Y., Lopez de Alda M., Barceló D. (2006). Evaluation of commercial immunoassays for the detection of estrogens in water by comparison with high-performance liquid chromatography tandem mass spectrometry HPLC-MS/MS (QqQ). Anal. Bioanal. Chem..

[72] Hercegová A., Dömötörová M., Matisová E. (1153). Sample preparation methods in the analysis of pesticide residues in baby food with subsequent chromatographic determination. J. Chromatogr. A.

[73] Rodriguez-Mozas S., Lopez de Alda M., Barceló D. (1152). Advantages and limitations of on-line solid phase extraction coupled to liquid chromatography-mass spectrometry technologies *versus* biosensors for monitoring of emerging contaminants in water. J. Chromatogr. A.

[74] Pawliszyn J., Lord H. (2010). Reference. Handbook of Sample Preparation.

[75] Fatoki O.S., Awofolu R.O. (2003). Methods for selective determination of persistent organochlorine pesticide residues in water and sediments by capillary gas chromatography and electron-capture detection. J. Chromatogr. A.

[76] Xue N., Xu X., Jin Z. (2005). Screening 31 endocrine-disrupting pesticides in water and surface sediment samples from Beijing Guanting reservoir. Chemosphere.

[77] Landrigan P.J., Claudio L., Markowitz S.B., Berkowitz G.S., Brenner B.L., Romero H., Wetmur J.G., Matte T.D., Gore A.C., Godbold J.H., Wolff M.S. (1999). Pesticides and inner-city children: Exposures, risks, and prevention. Environ. Health Perspect..

[78] Húšková R., Matisová E., Hrouzková S. (2010). Mass spectrometry with negative chemical ionization and its use in GC-MS analysis of organic pollutants. Chem. Listy.

[79] (2009). Tuija Pihlström. Method Validation and Quality Control Procedures for Pesticide Residues Analysis in Food and Feed; Document No. SANCO/10684/2009.

[80] Kotretsou S.I., Koutsodimou A. (2006). Overview of the applications of tandem mass spectrometry (MS/MS) in food analysis of nutritionally harmful compounds. Food Rev. Int..

[81] Čajka T., Hajšlová J. (1058). Gas chromatography-high-resolution time-of-flight mass spectrometry in pesticide residue analysis: Advantages and limitations. J. Chromatogr.A.

[82] Van Hoeck E., Canale F., Cordero C., Compernolle S., Bicchi C., Sandra P. (2008). Multiresidue screening of endocrine-disrupting chemicals and pharmaceuticals in aqueous samples by multi-stir bar sorptive extraction-single desorption-capillary gas chromatography/mass spectrometry. Anal. Bioanal. Chem..

[83] Ostrea E.M., Bielawski D.M., Posecion N.S., Corrion M., Villanueva-Uy E., Bernardo R.C., Jin Y., Janisse J.J., Ager J.W. (2009). Combined analysis of prenatal (maternal hair and blood) and neonatal (infant hair, cord blood and meconium) matrices to detect fetal exposure to environmental pesticide. Environ. Res..

[84] Maštovská K., Lehotay S.J. (1000). Practical approaches to fast gas chromatography-mass spectrometry. J. Chromatogr. A.

[85] Hrouzková S., Matisová E., Stoytcheva M. (2011). Fast Gas Chromatography and Its Use in Pesticide Residues Analysis. Pesticides-Strategies for Pesticides Analysis.

[86] Donato P., Tranchida P.Q., Dugo P., Dugo G., Mondello L. (2007). Rapid analysis of food products by means of high speed gas chromatography. J. Sep. Sci..

[87] Klee M.S., Blumberg L.M. (2002). Theoretical and practical aspects of fast gas chromatography and method translation. J. Chromatogr. Sci..

[88] Dömötörová M., Kirchner M., Matisová E., de Zeeuw J. (2006). Possibilities and limitations of fast GC with narrow-bore columns. J. Sep. Sci..

[89] Kirchner M., Matisová E., Otrekal R., Hercegová A., de Zeeuw J. (1084). Search on ruggedness of fast gas chromatography-mass spectrometry in pesticide residues analysis. J. Chromatogr. A.

[90] Cramers C.A., Leclercq P.A. (1999). Strategies for speed optimisation in gas chromatography: An overview. J. Chromatogr. A.

[91] De Zeeuw J., Peene J., Jansen H.G., Lou X.W. (2000). A simple way to speed up separations by GC-MS using short 0.53 mm columns and vacuum outlet conditions. J. High Res. Chromatogr.

[92] Kirchner M., Matisová E. (2005). Present state and perspectives of fast GC-MS application. Chem. Listy.

[93] Kirchner M., Matisová E., Hrouzková S., de Zeeuw J. (1090). Possibilities and limitations of quadrupole mass spectrometric detector in fast gas chromatography. J. Chromatogr. A.

[94] Anastassiades M., Maštovská K., Lehotay S.J. (1015). Evaluation of analyte protectants to improve gas chromatographic analysis of pesticides. J. Chromatogr. A.

